# Novel Insights into Parkin–Mediated Mitochondrial Dysfunction and “Mito-Inflammation” in α-Synuclein Toxicity. The Role of the cGAS–STING Signalling Pathway

**DOI:** 10.2147/JIR.S468609

**Published:** 2024-07-11

**Authors:** Magdalena Gąssowska-Dobrowolska, Gabriela Olech-Kochańczyk, Carsten Culmsee, Agata Adamczyk

**Affiliations:** 1 Department of Cellular Signalling, Mossakowski Medical Research Institute, Polish Academy of Sciences, Warsaw, Poland; 2 Institute of Pharmacology and Clinical Pharmacy, University of Marburg, Marburg, Germany; 3 Center for Mind Brain and Behavior – CMBB, University of Marburg, Marburg, Germany

**Keywords:** α-synuclein, Parkin, mtDAMPs, mito-inflammation, sterile inflammation, cGAS–STING pathway

## Abstract

The prevalence of age-related neurodegenerative diseases, such as Parkinson’s disease (PD) and related disorders continues to grow worldwide. Increasing evidence links intracellular inclusions of misfolded alpha-synuclein (α-syn) aggregates, so-called Lewy bodies (LB) and Lewy neuritis, to the progressive pathology of PD and other synucleinopathies. Our previous findings established that α-syn oligomers induce S-nitrosylation and deregulation of the E3-ubiquitin ligase Parkin, leading to mitochondrial disturbances in neuronal cells. The accumulation of damaged mitochondria as a consequence, together with the release of mitochondrial-derived damage-associated molecular patterns (mtDAMPs) could activate the innate immune response and induce neuroinflammation (“mito-inflammation”), eventually accelerating neurodegeneration. However, the molecular pathways that transmit pro-inflammatory signals from damaged mitochondria are not well understood. One of the proposed pathways could be the cyclic GMP-AMP synthase (cGAS) – stimulator of interferon genes (STING) (cGAS–STING) pathway, which plays a pivotal role in modulating the innate immune response. It has recently been suggested that cGAS–STING deregulation may contribute to the development of various pathological conditions. Especially, its excessive engagement may lead to neuroinflammation and appear to be essential for the development of neurodegenerative brain diseases, including PD. However, the precise molecular mechanisms underlying cGAS–STING pathway activation in PD and other synucleinopathies are not fully understood. This review focuses on linking mitochondrial dysfunction to neuroinflammation in these disorders, particularly emphasizing the role of the cGAS–STING signaling. We propose the cGAS–STING pathway as a critical driver of inflammation in α-syn-dependent neurodegeneration and hypothesize that cGAS–STING–driven “mito-inflammation” may be one of the key mechanisms promoting the neurodegeneration in PD. Understanding the molecular mechanisms of α-syn–induced cGAS–STING–associated “mito-inflammation” in PD and related synucleinopathies may contribute to the identification of new targets for the treatment of these disorders.

## Introduction

Parkinson’s disease (PD) is a widespread, age-related, fatal neurodegenerative disorder that impairs movement and is accompanied by dementia, affecting 3% of the global population over the age of 65.^1^ The most prominent neuropathological hallmarks of PD are the presence of intracellular inclusions containing misfolded α-synuclein (α-syn) aggregates, such as Lewy bodies (LB) and Lewy neuritis, and progressive dopaminergic neurodegeneration in the *substantia nigra pars compacta* (SNpc). Aggregated forms of α-syn have also been implicated in other neurodegenerative diseases such as dementia with Lewy bodies (DLB), multiple system atrophy (MSA), pure autonomic failure (PAF), and REM sleep behaviour disorder (RBD), collectively termed α-synucleinopathies.^2^ Although the discovery of the critical role of this protein in the pathogenesis of PD is almost thirty years old, many fundamental questions regarding successful strategies to target α-syn toxicity to prevent neurodegeneration remain unanswered. Proposed mechanisms of α-syn neurotoxicity include oxidative/nitrosative stress generation, mitochondrial and proteasomal dysfunction as well as neuroinflammation, but links between these detrimental processes are poorly understood.^3^,^4^ Our previous studies show that α-syn–induced nitrosative stress leads to S-nitrosylation and inactivation of another PD-associated protein, an E3 ubiquitin ligase, Parkin,^5^,^6^ and that α-syn–evoked Parkin down-regulation promotes mitochondrial dysfunction in neuronal cells.^7^ Parkin regulates protein breakdown by the ubiquitin–proteasome system and is essential for mitochondrial quality control (MQC) assurance.^8^ Upon loss of the mitochondrial membrane potential, Parkin is translocated to the mitochondrial surface and promotes degradation of the defective organelle by mitophagy, a selective type of autophagy.^9^ Therefore, Parkin could be a “multipurpose neuroprotective agent” that protects mitochondrial integrity and prevents mitochondrial stress–induced inflammation, oxidative stress, and cell death.^10–18^ In addition, Parkin can mitigate neuroinflammation through interaction with several key immune regulatory pathways, including those involving the nucleotide-binding oligomerization domain (NOD)-like receptor (NLR) family pyrin domain containing protein 3 (NLRP3) inflammasome, nuclear factor κB (NF-κB) and tumour necrosis factor-α (TNF-α) receptor (TNFR) – associated factors.^19–21^ The effects of Parkin deficiency, combined with mitochondrial damage and release of mitochondrial-derived damage-associated molecular patterns (mtDAMPs), may induce the abnormal activation of NLRP3 and its signalling mediator, cyclic GMP-AMP synthase (cGAS) – stimulator of the interferon genes (STING) (cGAS–STING) immune pathway.^22–24^ This could render neurons extremely vulnerable to any additional mitochondria-targeting insults, effectively accelerating their degeneration. Overall, defects in Parkin–mediated mitophagy, resulting mitochondrial stress, and the accumulation of damaged mitochondria may contribute to PD. The involvement of Parkin in PD-associated inflammation has been confirmed in mouse models.^11^,^25–27^ Moreover, it was recently shown that mitochondrial damage linked to mutations in quality control proteins such as the PD-associated PTEN–induced putative kinase 1 (PINK1)/Parkin activated the DNA–sensing cGAS and its signaling effector– STING, thus leading to an inflammatory response.^28^,^29^ As an essential component of the innate immunity, the cGAS–STING pathway is pivotal in activating the inflammatory response.^30^ Beyond its involvement in host defence against infectious microbes (as cGAS–STING has emerged as a key player against pathogens by detecting cytosolic foreign DNA and prompting a robust innate immune response),^31^ cGAS is also the main sensor that detects self-DNA, including genomic and mitochondrial DNA (mtDNA), released from either damaged, dead, or transformed cells.^32^ Upon mitochondrial damage, mtDNA is released into the cytoplasmic matrix as a danger signal, triggering cGAS, which generates the 2′3′- cyclic GMP-AMP (2’3’-cGAMP or cGAMP) that then activates a STING–mediated innate immune response, culminating in the production of type I interferons (IFN-I) and several other inflammatory mediators such as pro-inflammatory interleukin-6 (IL-6) and TNF-α.^33–35^ After secretion, IFN-I acts through the type I interferon receptor/or interferon-α/β receptor (IFNAR) on target cells, stimulating the transcription of hundreds of interferon-stimulated genes (ISGs), including pro-inflammatory cytokine, that together assist in counteracting threats.^36^ Although innate immune activation and inflammatory response within the central nervous system (CNS) is a neuroprotective and desirable mechanism against a variety of pathogens, injuries and non-infectious stressors, prolonged and uncontrolled inflammation can result in tissue damage and disease.^37^,^38^ Growing evidence has indicated that excessive engagement of the cGAS–STING signaling pathway plays a role in various pathological processes.^31^ Inappropriate activation of the cGAS–STING triggered by the nuclear DNA, mtDNA, mitochondrial damage and failures in Parkin–mediated mitophagy has been linked to neurodegenerative diseases.^31^,^39^,^40^ Aberrant cGAS–STING response contributes to neuroinflammation and promotes degeneration of neuronal cells.^41^ In chronic neurodegenerative diseases states, exacerbated cGAS–STING–mediated IFN-I response shapes the glia phenotype and accelerates disease progression.^42^ Aberrant overstimulation of this highly versatile innate immune sensing system has recently been linked to the aetiology of PD.^43–45^ Inflammation may enhance progression of PD pathology, but the relationships between α-syn accumulation, Parkin deregulation, and detrimental inflammatory responses are not fully elucidated. Our main hypothesis discussed here is that α-syn–induced Parkin dysfunction disturbs the mitochondrial network homeostasis, leading to mitochondrial stress, cGAS–STING pathway activation and neuroinflammation (“mito-inflammation”), thereby promoting the progressive neurodegeneration in PD. We suggest that the cGAS–STING signalling pathway is a key trigger in α-syn–induced “mito-inflammation”, which could be one of the pivotal mechanisms for the initiation and spread of PD pathology.

## Parkin as a Regulator of Mitochondrial Function

Mitochondria are pivotal organelles for many cellular functions and are the primary energy-generating system in most eukaryotic cells. The architecture of mitochondria is essential for their proper function and also for the confinement of mitochondria-derived immunogenic molecules.^46^ In addition to their role in cellular energy production in the form of adenosine triphosphate (ATP), mitochondria are implicated in the regulation of programmed cell death, calcium homeostasis and many other crucial physiological cellular processes.^47^,^48^

The disruption of mitochondrial homeostasis is a frequently reported key feature in ageing organisms and a major hallmark in neurodegenerative diseases, including Alzheimer’s, Huntington’s disease, and amyotrophic lateral sclerosis (ALS).^49^ These organelles are also important players in the pathophysiology of PD. Particularly, failure of MQC and the accumulation of damaged mitochondria in neurons along with the release of mtDAMPs are proposed to be important drivers of PD pathology.^19^,^21^,^50^ MQC includes a set of processes spanning from mitochondrial biogenesis to plasticity controlled by coordinated cycles of fusion and fission, autophagy, and quality control mechanism called the mitochondrial unfolded protein response (UPR^mt^) that are intended to ensure cellular and organismal homeostasis.^51^,^52^ A key player in orchestrating mitochondrial quality assurance across various tissues is Parkin, an E3 ubiquitin ligase, also known as Parkinson Disease Protein 2 (PARK2). Parkin is implicated in autophagic clearance of damaged mitochondria, regulation of mitochondrial fusion and fission dynamics, and in protecting mitochondrial biogenesis.

Mitophagy, the selective macroautophagy of mitochondria, can be triggered by various stimuli such as the loss of mitochondrial membrane potential,^8^ the presence of unfolded proteins in the mitochondrial matrix,^53^ or an altered protein composition of the mitochondrial outer membrane.^54^,^55^ Parkin plays a pivotal role in initiating mitophagy^56–59^ and this process is assisted by the PINK1 kinase, crucial for the recruitment of Parkin to the mitochondrial outer membrane of damaged mitochondria.^58^,^60^,^61^ Notably, mutations leading to the loss of PINK1 and Parkin function are the predominant causes of autosomal recessive early PD,^62^,^63^ characterised by a relatively benign course and positive responsiveness to dopamine replacement therapy.^64^

Under physiological conditions, by interacting with translocase complexes of the outer and inner mitochondrial membrane, PINK1 is constitutively translocated onto polarised mitochondria,^65^ where it is cleaved and then undergoes degradation in the proteasome.^66^ However, under pathological conditions associated with reactive oxygen species or the loss of mitochondrial membrane potential, PINK1 undergoes autophosphorylation, dimerisation, and rapid accumulation on the outer membrane of damaged or uncoupled mitochondria.^59^,^67^ Subsequently, PINK1 phosphorylates mitofusin 2 (Mfn2), enabling it to act as a mitochondrial outer membrane receptor for Parkin.^68^ PINK1’s multifaceted role includes the activation of Parkin not only by controlling its subcellular location, but also by phosphorylating its Ser65,^69–73^ thereby abolishing Parkin’s auto-inhibition.^74^ Furthermore, PINK1 phosphorylates ubiquitin,^75–78^ influencing the assembly of polyubiquitin chains and their susceptibility to hydrolysis.^79^ Critically, not only mitofusins but also phosphorylated polyubiquitin chains on the mitochondrial surface facilitate Parkin tethering,^80–82^ creating in this way a positive regulatory loop^78^ that amplifies the pro-mitophagic signaling initiated by PINK1.^83^

Activated Parkin primarily functions by ubiquitin-tagging proteins of damaged mitochondria, including mitofusin 1 (Mfn1), Mfn2, dynamin-related protein 1 (Drp1), mitochondrial fission protein 1 (Fis1) and voltage-dependent anion channel 1 (VDAC1).^55^,^78^,^84^ These proteins are directed to degradation in the ubiquitin–proteasome system, remodelling the composition of the mitochondrial outer membrane.^54^,^55^ Before degradation, these proteins may recruit two receptors possessing a ubiquitin-binding domain, nuclear dot protein 52 (NDP52) and optineurin (OPTN), that canonically initiate selective mitophagy.^83^ Notably, the affinity of OPTN towards ubiquitin chains increases upon its phosphorylation by TANK-binding kinase 1 (TBK1), which in turn has been shown to be activated by mitochondrial damage in a PINK1- and Parkin-dependent manner.^83^ Both NDP52 and OPTN initiate phagophore formation by recruiting a protein complex containing Unc-51-like kinase 1 (ULK1), the principal driver of autophagy. ULK1 is activated by AMP-activated protein kinase (AMPK) upon the drop of cellular ATP and inhibited by mammalian target of rapamycin (mTOR) complex 1 (mTORC1).^85^ AMPK-activated ULK1 rapidly phosphorylates conserved Ser108 of Parkin,^86^ which in turn can interact with and ubiquitinate mTOR kinase, the main component of mTORC1.^87^ Moreover, Parkin is able to interact with autophagy and beclin 1 regulator 1 (AMBRA1), another driver of autophagy,^88^ and is also involved in a non-canonical mechanism of mitochondrial quality assurance, in which vesicles budding off the oxidatively stressed mitochondria are ultimately directed to the lysosome.^89^,^90^ Deubiquitinases, which counteract the ubiquitin ligase activity of Parkin, remove Parkin-attached ubiquitin from damaged mitochondria, preventing induction of excessive mitophagy.^91^,^92^ Also, Parkin–mediated ubiquitination of Mfn2 eventually suppresses mitophagy,^68^ likely implementing a self-restraining mechanism, which prevents exaggerated mitochondrial elimination. Mfn1 also undergoes ubiquitination–mediated degradation.^54^,^93–95^

Moreover, the PINK1/Parkin pathway influences mitochondrial function through the control of their dynamics. Proper mitochondrial functioning relies on a balance between fusion and fission processes, and disruption of which impairs the entire mitochondrial network. Respiratory active cells exhibit mitochondrial fusion, while resting cells often have fragmented mitochondria.^96^ Oxidative stress alters mitochondrial dynamics, with moderate stress promoting fusion,^97^ whereas severe stress induces excessive fragmentation^98^ leading to decreased membrane potential, decreased ATP levels, increased mitochondrial free radicals, and cell death. In rat hippocampal and dopaminergic neurons, PINK1 or Parkin overexpression tilts the fusion–fission balance toward fission, whereas inactivation of PINK1 shifts it in the reverse direction.^99^ In mammals, mitochondrial fusion involves proteins anchored in the outer mitochondrial membrane, specifically, Mfn1 and Mfn2 from the mitofusin family, and the dynamin-like GTPase localised to mitochondrial inner membrane, optical atrophy 1 (Opa-1) protein. Under severe stress, Parkin is involved in ubiquitinating mitofusin family proteins, inhibiting the fusion of damaged mitochondria.^93^,^95^,^100–102^ This ubiquitination leads to mitochondrial network fragmentation, facilitating the removal of malfunctioning mitochondria. Drp1 is a cytoplasmic protein crucially involved in the mitochondrial fission process. Some adapter proteins, including mitochondrial fission factor (Mff), facilitate Drp1’s interaction with Fis1 on the outer mitochondrial membrane. Parkin interferes with this interaction by ubiquitinating Drp1, eventually preventing the fission of functional mitochondria.^103^

The generation of new mitochondria is as crucial as the removal of damaged ones. Mitochondrial biogenesis is tightly regulated by several members of the peroxisome proliferator-activated receptor gamma coactivator 1 (PGC-1) family of transcription factors, including PGC-1α, PGC-1β, and PGC-1-related coactivator (PRC).^104^ Parkin plays a pivotal role in regulating mitochondrial biogenesis by ubiquitinating and targeting to degradation the transcription factor Parkin-interacting substrate (PARIS) that inhibits the expression of PGC-1α.^105^ The control of PARIS and its clearance by Parkin is orchestrated by PINK1, which phosphorylates PARIS on two residues, Ser322 and Ser613.^106^ The knockout of Parkin in the adult mouse ventral midbrain leads to defective mitochondrial biogenesis.^107^ In line with this regulatory mechanism, Parkin silencing significantly reduces the level of PGC-1α in PC12 cells.^7^ The abrogation of Parkin function in a mouse model of age-related sporadic PD coincides with increased PARIS levels and reduced PGC-1α signalling.^108^ Recently, PARIS has been implicated in the suppression of nuclear factor-erythroid 2 related factor 2 (NRF2)–driven transcription,^109^ suggesting that Parkin may also be essential for appropriate cellular responses to oxidative stress.

## Relationship Between Mitochondrial Dysfunction and Neuroinflammation – the “Mito-Inflammation” Concept

Both mitochondrial dysfunction and chronic neuroinflammation are strictly involved in neurodegeneration and pathogenesis of neurodegenerative diseases, although the underlying mechanisms engaged in neuronal death have not been fully explained.^39^,^110^,^111^ New data indicate a relationship between mitochondrial pathology and neuroinflammation. In particular, defective MQC together with mtDAMPs generation are proposed to be major contributors to the pathogenic mechanisms of neuronal degeneration.^112^ The plethora of evidence strongly suggests that mitochondrial integrity and innate immunity are closely interlinked.^113–115^ Mitochondrial dysfunction may precede neuroinflammation and act as an inflammation-promoting agent during neurodegenerative disease progression.^116^ In turn, chronic inflammation may promote and exacerbate mitochondrial damage, thus generating a vicious cycle of neurotoxic events. This vicious cycle leads to the release of mtDAMPs and activates specific inflammatory cascades.^111^ Therefore, an in-depth understanding of the potential molecular mechanisms of the interaction between mitochondrial dysfunction and neuroinflammation as well as signalling pathways leading to mitochondria–induced inflammation (“mito-inflammation”) in neurodegenerative disorders may prove helpful in identifying new therapeutic targets and approaches for the treatment of neurodegenerative diseases, especially PD.

### mtDAMPs - Key Mediators Linking Mitochondrial Dysfunction to Neuroinflammation

Upon deleterious stimulus, compromising the integrity of the mitochondrial membrane results in the release of some molecules that normally reside inside the mitochondria.^39^,^110^ These released molecules, often referred to as mtDAMPs (also known as mitochondrial alarmins),^117^,^118^ act as a stress signal for the body, activating innate immune system receptors and downstream signalling engaged in “sterile inflammation” and associated pathology, according to a theory developed by Matzinger in 2002.^119^,^120^ The endosymbiotic origin of mitochondria^121^ causes the immune system to erroneously recognise mtDAMPs as bacteria and triggers an innate immune response.^122^ Therefore, in light of the endosymbiotic theory, mitochondria are the main regulators of innate immunity.^123^

The innate immune system, as a first line of defence against various pathogens, injury, and stress, deploys germline-encoded receptors named pattern-recognition receptors (PRRs) to detect pathogens and danger signals through the recognition of conserved molecular motifs, called pathogen-associated molecular patterns (PAMPs) and damage-associated molecular patterns (DAMPs).^117^ PRRs that recognise and detect both pathogenic PAMPs and mitochondrial DAMPs are NLRs, cGAS, and toll-like receptors (TLRs) at the plasma membrane or endosomes.^34^ Most of the PRRs are expressed not only in specialised innate immune cells, such as macrophages, microglia, dendritic cells or neutrophils, but also in non-immune cells, including neurons.^34^ Although an inflammatory response within CNS, activated in response to infection, toxic accumulation and other pathological injuries and non-infectious stressors, is a protective mechanism, excessive, prolonged and uncontrolled overactivation of glial cells can result in tissue damage and disease.^37^,^38^,^124^ Under pathological conditions, when mitochondrial malformations are uncontrolled and/or damaged mitochondria cannot be properly removed by mitophagy, mtDAMPs are released into the cytoplasm or out of the cell leading to abnormal activation of the PRRs and the innate immune system. This, in turn, enhances neuroinflammatory processes.^112^,^125^,^126^ Long-term overactivated glial cells cooperate, overproduce and release various harmful pro-inflammatory, stress-inducing factors, which aggravate mitochondrial damage, cause a gradual loss of their function and form a vicious cycle of mitochondrial dysfunction and neuroinflammation. The interactions between mitochondrial dysfunction and neuroinflammation ultimately result in neurodegenerative diseases. Among the mitochondrial DAMPs, considered as danger signals, the pro-inflammatory molecules that induce and exacerbate the inflammatory response within CNS include inter alia: mtDNA, mitochondrial-derived reactive oxygen species (mtROS), ATP, mitochondrial transcription factor A (TFAM), cardiolipin, and cytochrome C.^39^,^127–133^

#### mtDNA

In response to certain cellular stress or environmental insults, mtDNA, a key signalling molecule that regulates energy production and cell metabolism, can be released from damaged organelles into the cytoplasm or even outside the cell, where it engages various PRRs to trigger an immune response and activate pro-inflammatory pathways.^39^,^134^ There are several potential routes by which mtDNA is released into the cytosol to mediate pro-inflammatory responses.^42^ However, the exact mechanisms of mtDNA entry into the cytosol are not well elucidated. Likewise, little is known about the mechanism responsible for the loss of mitochondrial inner membrane integrity.

The protein that organises mtDNA into nucleoids (discrete protein–DNA complexes protecting mtDNA against oxidative damage) and regulates its segregation and number is the mitochondrial DNA binding protein, TFAM.^135^ A haplodeficiency of TFAM via disorganisation of the mitochondrial genome and mitochondrial stress leads to the release of mtDNA into the cytosol, which elicits the expression of several ISGs in a cGAS–STING pathway activation-dependent manner.^136^,^137^ Under physiological conditions, an mtDNA-binding TFAM is localised in the mitochondrial inner membrane. However, when mitochondria are damaged due to conditions of stress, the combination of caspase and B-cell lymphoma-2 (Bcl-2) inhibitors induces the translocation of this protein to the cytosol, where it assists cGAS in sensing cytosolic DNA.^42^,^136^ Studies show that loss of TFAM causes a three-to-four-fold rise in mtDNA in the cytosol associated with activation and an increase in the expression of more than 39 ISGs.^42^ Additionally, knocking down cGAS, STING, TBK1 or interferon regulatory factor 3 (IRF3) in the TFAM^±^ cells leads to the suppression of the ISGs, overall confirming that TFAM depletion–induced mtDNA stress involves the cGAS–STING pathway.^42^,^135^

The proposed mechanism of mtDNA leakage involves the participation of apoptosis regulator, Bcl-2-associated X (BAX) and Bcl-2 antagonist/killer 1 (BAK1) protein.^136^,^138^ Under conditions of extreme stress, activation of BAX/BAK induces the formation of extremely large macropores in the outer mitochondrial membrane. These progressively widen, allowing the extrusion/herniation of the inner mitochondrial membrane into the cytosol, driven by the increase in osmotic pressure in the mitochondrial matrix. Formation of these macropores in the outer membrane leads to permeabilisation of the inner mitochondrial membrane and a leak of mitochondrial macromolecules into the cytosol, including mtDNA.^139^ Another theory assumes the escape of mtDNA to the cytoplasm via the VDAC, where VDAC oligomers can form large pores on the outer mitochondrial membrane under mild-stress conditions.^140^ Other possible routes include the transit of mtDNA through the mitochondrial permeability transition pore (mPTP)^141^ or the formation of mitochondria-derived vesicles (MDVs), which transport mtDNA to the endosomal-lysosomal pathway or to the plasma membrane, as alternative routes for inner mitochondrial membrane traversal.^34^ The opening of mPTP allows the release of comparatively small mtDNA fragments. Thus, it has been proposed that mtDNA released through this pore is limited to fragments of sizes smaller than 700 bp.^142^,^143^

mtDNA derived from damaged mitochondria acts as a mtDAMP to regulate the inflammatory response. mtDNA potently triggers the innate immune response and its high immunogenic potential is due to the presence of unmethylated (or hypomethylated) CpG motifs within its structure. The motifs are similar to those of bacterial DNA and are recognised by PRRs.^34^,^144–146^

mtDNA modulates the inflammatory response by triggering Toll-like receptor 9 (TLR9),^39^ cytosolic inflammasomes,^114^,^147^,^148^ and stimulation of ISGs transcription.^117^ mtDNA, containing an unmethylated CpG-DNA sequence, is a ligand for endosomal TLR9 and can mediate pro-inflammatory response via the NF-κB pathway. A downstream signalling cascade includes myeloid differentiation primary response 88 (MyD88), which activates the p38 mitogen-activated protein kinase (MAPK) and finally triggers the nuclear transcription factor NF-κB signalling to promote pro-inflammatory immune response.^149–151^

Released from damaged mitochondria into the cytoplasm, mtDNA can also activate the NLRP3 inflammasome, promoting the cleavage of pro-interleukins into mature interleukin-1β (IL-1β), interleukin-18 (IL-18) by activating caspase-1 subunit of the NLRP3 complex, thereby inducing inflammation.^147^,^152^ NLRP3 is the best-characterised inflammasome and one of the most involved in mtDNA sensing.^117^ In particular, oxidised mtDNA preferentially activates NLRP3.^147^ Interestingly, Nakahira and Zhou discovered that activated NLRP3 can also increase cytosolic mtDNA release, and this was mediated through altered Parkin function and impaired mitophagy,^153^ which means that NLRP3 stimulation could play a role as a positive feedback loop, and further exacerbate the pro-inflammatory response.^114^,^154^ It is suggested that NLRP3 is one of the key factors responsible for the initiation and progression of chronic inflammation.

Another well-described cytoplasmic mtDNA sensor, and innate immune receptor, is the AIM2 inflammasome, also known as absent in melanoma-2. It belongs to the IFI20X-IFI16 (PYHIN) protein family and binds to DNA via hematopoietic interferon-inducible nuclear proteins. AIM2 responds preferentially to double-stranded DNA (dsDNA) and non-oxidised DNA released from damaged host cells, causing the gasdermin D–mediated secretion of the bioactive effectors IL-1β and IL-18, as well as triggering pyroptotic cell death, thereby driving the progression of “sterile inflammatory” diseases.^155^,^156^

mtDNA released into the cytoplasm by defective and damaged mitochondria also causes the activation of the DNA–induced cGAS–STING pathway and the promotion of neuroinflammation.^135^ mtDNA damage and high levels of mtDNA fragments circulating in plasma positively correlate with chronic inflammation and oxidative stress, suggesting an important role of free mtDNA as a mtDAMP and stimulators of the cGAS–STING–TBK1 signalling cascade.^157^ Thus, circulating mtDNA is increasingly recognised as a key mediator linking mitochondrial dysfunction to inflammation.^39^

#### Other mtDAMPs

Besides mtDNA, mitochondria also contain other pro-inflammatory molecules classified as mtDAMPs, which are released from damaged mitochondria and amplify the pro-inflammatory response. Among them, we distinguish mitochondrial-derived mtROS, an upstream mediator required for NLRP3 inflammasome activation.^130^ Mainly produced by the mitochondrial electron transport chain (ETC) and mitochondrial NADPH oxidase (NOX), mtROS are the major source of oxygen radicals, including superoxide (O_2_^•−^), nitric oxide (NO), hydrogen peroxide (H_2_O_2_), and singlet oxygen.^116^ The imbalance between mtROS generation and their removal, due to overproduction of ROS and/or decreased activity of the antioxidant defence system, results in oxidative stress, which leads to oxidative damage and mitochondrial destruction.^158^ Mitochondrial dysfunction and exacerbation of mtROS levels have been proposed to contribute to “mito-inflammation”. Excessive mtROS levels sustain and intensify inflammation causing tissue damage and becoming a chronic phenomenon in pathological conditions.^116^ mtROS may trigger the stimulation of pro-inflammatory signalling through activation of the redox-sensitive transcription factors NF-κB,^159^,^160^ hypoxia-inducible factor 1 (HIF-1)^161^ and activator protein 1 (AP-1).^162^ As well as inducing the expression of inflammasome genes, such as *Nlrp3, Nlrc4*, and *Il-1b* genes. mtROS accumulation acts destructively on the mtDNA particularly susceptible to the effect of mtROS due to the lack of protective histones and effective complex mechanism to repair DNA.^39^ Synergistic stimulation between the inflammasome and redox-vulnerable inflammation reinforces the inflammatory response.^163^ NLRP3 facilitates mPTP opening, thereby contributing to the release of mtDNA.^114^ In this manner, NLRP3 activation could act as an upstream checkpoint of the innate immune system during the deployment of “sterile inflammation”, involving the DNA-sensing cGAS–STING pathway. Hence, a self-sustaining circle of events involving loss of mitochondrial function, ROS bursts, and mtDNA damage caused by NLRP3 activators has been proposed.^147^ Oxidised mtDNA may serve as the ultimate NLRP3 ligand,^147^ thus confirming the crucial role played by ROS in inflammasome activation.^154^

Similarly, extracellular ATP, released by the Pannexin 1 (Panx1) channel from injured or dying mitochondria under stress conditions,^164^ serves as a mtDAMP and may activate the caspase-1 signalling cascade mediated by NLRP3 through the purinergic P2X7 receptor of glial cells, leading to neuroinflammation.^118^,^165^ P2X7 receptors are preferentially located on microglia, and their activation is associated with inflammation.^166^ The binding of extracellular ATP to the P2X7 receptor leads to the opening of this cation channel that allows K^+^ efflux from and Ca^2+^ and Na^+^ influx into the cells. Calcium influx into the cell triggers several intracellular signalling cascades, resulting in the activatory cleavage of caspase-1, which in turn catalyses the formation of mature IL-1β and IL-18 from their biologically inert propeptides.^117^ Moreover, ATP–induced activation of the P2X7 receptor leads to the stimulation of the nuclear factor of activated T-cells (NFAT) and MAPK cascades, also known as protein kinase C/mitogen-activated protein kinase (PKC/MAPK) pathways, leading to the production and release of C-X-C Motif Chemokine Ligand 2 (CXCL2) in microglia and exacerbating inflammation.^167^

It has been reported that cardiolipin externalisation and relocation to the surface of dysregulated mitochondria also mediates the activation of NLRP3 to initiate a caspase-1-dependent neuroinflammatory response and release bioactive IL-18 and IL-1β.^130^ Cardiolipin, tetra-acylated diphosphatidylglycerol lipid, primarily exists in the mitochondria as a component of the mitochondrial inner membrane and is indispensable in maintaining the electron transport chain.^118^,^168^ However, under stress conditions, mitochondrial damage and depolarisation result in the translocation of cardiolipin to the outer mitochondrial membrane. Attachment of cardiolipin to NLRP3 activates the inflammasome, resulting in a neuroinflammatory response.^130^ Depending on the amount, and level of cardiolipin saturation and oxidation it can exert both pro-mitochondrial or pro-apoptotic signals affecting mitochondrial function and inflammatory response.^169^ Exposed cardiolipin mediates the degradation of damaged mitochondria and their phagocytosis from the extracellular milieu, thus serving as an “eat-me” signal for eliminating defective mitochondria.^170^ Cardiolipin in the inner mitochondrial membrane acts as an anchor for cytochrome C. Oxidation of cardiolipin and its exposure on the outer mitochondrial membrane induce cytochrome C release and apoptosis.^171^

Cytochrome C is a 15-kDa water-soluble mitochondrial haemoprotein that can act also as mtDAMP when released into the extracellular space from damaged cells to activate neuroinflammation.^172^ Released from damaged mitochondria to the outside cytochrome C activates the mitogen-activated protein kinase/c-Jun N-terminal kinase (MAPK/JNK) signalling cascade by interacting with glial TLR4 receptors to regulate the function of immune cells in the brain.^132^,^133^ Damaged glial cells are a potential source of extracellular cytochrome C in the CNS.^39^ Cytochrome C causes immune activation of both microglia and astrocytes in a TLR4-dependent manner.^132^,^133^ In addition, extracellular cytochrome c may display direct pro-inflammatory properties mediated by the activation of NF-κB and causing neutrophil and monocyte-triggered inflammation.^173^

TFAM is a member of the high mobility group box (HMGB) family of proteins and acts as a guardian of the mitochondrial genome, playing important roles in mtDNA replication, maintenance and transcription.^118^ The correct expression of TFAM is crucial for the functioning of mitochondria and, consequently, cellular homeostasis. The presence of extracellular TFAM is described as inducing an inflammatory response, similar to the action of another important member of mtDAMPs, including HMGB1.^165^ When released externally from neurons or glia, TFAM can activate the receptor for advanced glycation endproducts (RAGE) or TLR4 on the plasma membrane surface of the glia, thus initiating the NF-κB–mediated nuclear signalling cascade to activate a pro-inflammatory response.^39^,^129^ TFAM also exacerbates the immunogenicity of mtDNA.^46^ TFAM, bound to mtDNA, can interact with the plasma membrane receptor RAGE and induce the internalization of mtDNA, thereby promoting its recognition by TLR9 and amplifying TNF-α release from TLR9-expressing plasmacytoid dendritic cells (pDCs).^174^,^175^

Despite the existence of many safeguards that prevent mitochondria from triggering harmful DAMPs-associated inflammatory reactions, when the homeostatic capacity of such systems is exceeded or when these systems become damaged, inflammatory reactions induced by mitochondria may become pathogenic and contribute to the aetiology of neurodegenerative diseases, including PD.^156^

### mtDAMPs-Dependent Activation of cGAS–STING Signalling Pathway

cGAS, a major sensor of cytoplasmic viral, retroviral, parasitic and bacterial DNA, as well as any dsDNA, including self-DNA (both genomic and mitochondrial DNA), is an innate immune system receptor, that undergoes a conformational change to an active state and generates the production of the second messenger molecule, cGAMP using ATP and guanosine triphosphate (GTP) as substrates.^176–178^ cGAS is a cytosolic nucleotidyltransferase, which is activated by DNA-inducing conformational changes around the catalytic active site of cGAS (encompassing its dimerisation, oligomerisation, and ultimately phase separation).^179^ The immune-stimulatory DNA-binding capacity of cGAS is contained in the less conserved, disordered, and containing a high density of positively charged residues in the N-terminal structural domain (1–160) of cGAS, while the highly conserved C-terminal catalytic domain (161–522) (composed of three DNA-binding sites, named “site A”, “site B”, and “site C”) is critical for the enzyme activity of cGAS and the synthesis of cGAMP.^180^,^181^ Recent studies have provided evidence that the Ser (13, 37, 64, 129 and 143) residues in the cGAS N-terminus are crucial for sensing genomic/chromatin DNA.^182^ In turn, three structures/sites in the catalytic part of cGAS suggest a sequential reaction mechanism in the cGAS-catalysed production of 2′3′- cGAMP.^183^ Site A is the primary site that mediates DNA binding–induced conformational changes of the activation loop in cGAS (rearranging the catalytic pocket of the enzyme to allow for optimal interaction with the substrates). DNA binding to the juxtaposed secondary B-site leads to the formation of the core 2:2 cGAS-DNA complex - the minimal active enzymatic unit. Site C provides an additional interaction between cGAS and DNA, contributing to the phase separation of the cGAS-DNA complex.^35^,^183^ Interestingly, structural studies uncovered mechanistic details of DNA binding to cGAS, providing explanations for the preferential response of human cGAS to DNA fragments longer than 45 bp and the enhanced activation of cGAS by TFAM-associated mtDNA.^135^,^184^ To ensure the activation of cGAS and its sufficient catalytic activity to lead to a robust induction of IFN-I transcription in vitro, an amount of a>50 mer of DNA is necessary.^185^,^186^ Thus, mechanistically, any dsDNA is bound by cGAS in a sequence-independent but length-dependent manner.^187^ The interactions are mostly mediated by positively charged residues in cGAS with a sugar-phosphate backbone of DNA, explaining the lack of sequence specificity of the interaction.^183^ Moreover, additional evidence for a DNA-length-dependent mechanism of cGAS activation was provided by the recent discovery of DNA–induced liquid-liquid phase separation (LLPS) of cGAS.^188^ The DNA–induced phase transition of cGAS, critically dependent on the concentration of both cGAS and DNA in the cell, promotes cGAMP production and innate immune signalling while providing a mechanism to avoid autoimmune reactions to low concentrations of self-DNA.^183^

cGAMP formed by cGAS subsequently binds to the adaptor protein STING. STING, also known as transmembrane protein 173 or MPYS, MITA, and ERIS, is a small (~40kDa) transmembrane protein located in the endoplasmic reticulum (ER) membrane.^180^ The binding of cGAMP to STING causes its profound and extensive conformational rearrangement that trigger a half-turn rotation, oligomerisation of STING dimers, and its liberation from anchoring factors (such as stromal interaction molecule 1 (STIM1)). The resulting active STING unit is capable of initiating effector functions. In the following steps, a prerequisite for the initiation of downstream signalling is the translocation of oligomerised STING from the ER through the ER–Golgi intermediate compartment (ERGIC) to the Golgi apparatus.^35^,^42^,^189–191^ So far, very little is known about the precise molecular events and the relationship between putative players involved in this trafficking process. It is only known that the canonical ER-to-Golgi transport machinery, composed of coatomer protein complex II (COPII) vesicles, relying on the GTPase SAR1A (secretion associated, Ras related GTPase 1A) and the COPII complex components, including SEC24-related protein C (SEC24C) as well as the ARF-GTPase ARF1, facilitates the STING translocation to the Golgi.^192^ Upon reaching the ERGIC and Golgi compartments, the C-terminal tail of STING recruits the downstream TBK1 through a conserved PLPLRT/SD amino acid binding motif, which promotes dimerisation–mediated TBK1 autophosphorylation of (Thr172) in the activation loop and thus TBK1 activation.^193^,^194^ In turn, activated TBK1 phosphorylates STING (at Ser366 in humans and Ser365 in mouse) in the C-terminal tail, providing a binding site for IRF3, thereby recruiting IRF3 for phosphorylation by nearby TBK1.^34^,^195^ A master regulator of IFN-I, IRF3, phosphorylated by TBK1 forms a dimer and translocates to the nucleus, where it turns on the expression of IFN-I.^35^,^196^

The STING oligomer translocated to the Golgi apparatus can also recruit and activate IκB kinase (IKK). This event results in the phosphorylation of IκBα (inhibitor of the NF-κB). Subsequent ubiquitination directs IκBα for its proteasomal degradation, resulting in the release of NF-κB heterodimers (comprising the p65 and p50 subunits) from inhibitory binding, and their subsequent translocation to the nucleus to up-regulate the production of pro-inflammatory cytokines and chemokines.^34^,^197–199^ Thus, induction of both downstream signalling pathways such as NF-κB signalling together with IRF3 activation, as a result of cGAS–STING stimulation, turns on robust transcription of IFN-I and a number of pro-inflammatory factors to enhance the innate immune response.^200^

After secretion, the IFN-I (interferon-alpha (IFN-α) and interferon-beta (IFN-β)) act on target cells through IFNAR, comprising interferon-α/β receptor alpha chain (IFNAR1) and interferon-α/β receptor beta chain (IFNAR2) subunits, associated with the Tyrosine kinase 2 (TYK2) and Janus kinase 1 (JAK1), respectively, and located on the target cells. Stimulation of TYK2 and JAK1 promotes activation of signal transducer and activator of transcription 1 and 2 (STAT1 and STAT2), which bind to the interferon regulatory factor 9 (IRF9), forming a heterotrimeric transcription factor complex termed IFN-stimulated gene factor-3 (ISGF3).^42^ By binding genes harbouring the interferon-sensitive response element (ISRE), ISGF3 complex leads to the stimulation of the transcription of hundreds of ISGs, including pro-inflammatory IL-1β and IL-6.^36^,^201^,^202^

Interferons are a group of cytokines, which are classified as type I (especially IFN-α and IFN-β), type II (INF-γ) and type III (INF-λ1–λ4 in humans).^203^ The brain is more vulnerable to the effects of IFNs than the peripheral tissues. IFN-I can directly change both the structural and functional integrity of neurons.^42^ Not only neurons but also microglia and astrocytes do express IFNAR and respond to IFN-I.^204^,^205^ The predominant type of cell which activates the cGAS–STING signalling pathway are the immunocompetent cells of the CNS, microglia.^204^,^205^ These tissue-resident macrophages, activated by danger signals, secrete IFN-I, which act on the IFNAR receptor on neurons, thereby eliciting defence mechanisms.^42^ In parallel, the astrocytes are also stimulated to alleviate inflammation.^42^ Activated microglia release a wide spectrum of pro-inflammatory cytokines, which act on astrocytes leading to their activation and the release of pro-inflammatory molecules. The activated glial cells cooperate and produce pro-inflammatory molecules (cytokines and chemokines), as well as ROS and reactive nitrogen species (RNS) (IL-6, IL-1β, TNF-α, iNOS, ROS, NO, ONOO-) to trigger an immune response.^38^ However, prolonged overactivation of glial cells together with the excessive engagement of this signalling system can cause chronic neuroinflammation contributing to neurodegeneration.^37^,^39^ Long-term overstimulation of the glia leads to the overproduction and release of various harmful stress-inducing factors that affect neurons and cause a gradual loss of their function.^124^,^206^ Moreover, the pro-inflammatory factors released by activated glia may aggravate mitochondrial damage, forming a vicious circle of toxic events. In chronic neurodegenerative states, aberrant cGAS–STING–induced generation of IFN-I shapes the microglia phenotype and accelerates disease progression.^207^

In addition to facilitating the inflammatory response, increasing evidence suggest that the cGAS–STING signalling pathway can induce and regulate various cell death pathways. In response to various stresses in dynamic microenvironments, it can stimulate the process of apoptosis,^208^ autophagy/mitophagy, necroptosis, pyroptosis, and ferroptosis.^209–212^

### From Mitochondrial Dysfunction Through cGAS–STING to “Mito-Inflammation” in PD

Among the pathogenic mechanisms of neurodegeneration in PD, the co-occurrence of mitochondrial dysfunction and neuroinflammation may be crucial to induce neuronal degeneration.^52^,^213^,^214^ In particular, defective mitochondrial quality control, mtDAMPs generation and activation of innate immune pathways, including cGAS–STING are proposed to be major contributing factors.^215^

The majority of PD-associated genes play a role in mitochondrial homeostasis.^216^,^217^ Mutations in the genes encoding PINK1 and/or PARKIN are linked to familial, autosomal recessive inherited forms of PD, providing evidence that the accumulation of damaged mitochondria may contribute to PD.^34^,^62^,^63^,^218^,^219^ Both the E3 ubiquitin ligase Parkin and PINK1 work together in the same cellular process, called mitophagy, thus playing pivotal roles in the clearance of damaged mitochondria.^42^,^220^ Disturbances of the mitophagy machinery may result in the accrual of damaged mitochondrial components, including mtDAMPs, which can be released into the cytosol or in the extracellular space and activate the innate immune receptors, which leads to stimulation of NF-κB, cGAS–STING pathway, NLRP3 inflammasome, and/or MAPK/JNK signalling cascade to promote inflammation, an important driver of PD pathology.^19^,^21^,^50^,^114^,^183^,^221–223^ The presence of mtDAMPs in circulating extracellular vesicles (EVs) has been described in older adults with PD and is characterised by a specific inflammatory signature.^224^ Interestingly, mitophagy was identified as a protective mechanism to limit the release of both mtROS and mtDNA from defective mitochondria.^34^,^114^,^154^ Mitophagy prevents the initiation of extensive inflammatory responses and favours the inhibition of inflammation driven by a small number of permeabilised, dysfunctional mitochondria (limited signalling via cGAS–STING and the NLRP3 promotes mitophagy, associated with the recruitment of Parkin to dysfunctional mitochondria, and TBK1-dependent phosphorylation of OPTN and consequent engulfment of mitochondria by formed autophagosomes).^156^

However, unrecoverable and uncontrolled massive dysfunction of the mitochondrion is associated with robust cGAS signalling and strong NLRP3 stimulation. This is accompanied by inhibition of mitophagy – as a consequence of caspase 1-dependent cleavage of Parkin elicited by robust NLRP3 activity, despite OPTN phosphorylation, which maximises fully-developed inflammatory responses.^153^ Thus, aberrant or inactivated mitophagy can lead to the accumulation of potent triggers of the innate immunity system response.^42^,^135^ Trinh et al have established that mutations in PINK1/Parkin increased the heteroplasmic mtDNA variants burden in blood, which was correlated with serum pro-inflammatory IL-6 levels.^225^

By linking impaired mitophagy and insufficient clearance of damaged mitochondria and inflammation, the release of mtDNA into the cytosol may trigger cGAS–STING–mediated activation of innate immunity.^226^ The cGAS–STING–driven inflammation has been implicated in neurodegeneration following mitophagy impairment. Increasing evidence suggests that mtDNA is a vector of neuroinflammation via the cGAS–STING axis in Parkin loss–mediated pathology.^227^ Midbrain neurons from patients with PD with biallelic Parkin mutations exhibited impaired mtDNA dynamics and increased cytosolic mtDNA levels. Loss of Parkin in the presence of mtDNA stress mirrored these phenotypes together with up-regulation of the cytosolic DNA sensor, cGAS protein.^228^ Furthermore, a recent study has also found that the depletion of PINK1, PARK9 and/or glucocerebrosidase beta 1 (GBA1) increased cytosolic dsDNA of mitochondrial origin and induced IFN-I responses and cell death in cellular models. In addition, interferon-inducible protein 16 (IFI16) (an innate immune sensor of intracellular DNA) and cytosolic dsDNA puncta of mitochondrial origin accumulate in the brains of patients with PD.^229^ Notably, IFI16 has the ability to the recruitment of STING and the production of IFN-I, or else forms the inflammasome with apoptosis-associated speck-like protein containing a C-terminal caspase recruitment domain (ASC) to induce the production of IL-1β and IL-18. Additionally, IFI16 activation stimulates the production of other cytokines and chemokines, such as IL-6, C-X-C Motif Chemokine Ligand 10 (CXCL10) and C-C Motif Chemokine Ligand 20 (CCL20).^230^ In a study conducted by Jimenez-Loygorri, pharmacological induction of PINK1/Parkin–mediated mitophagy curtailed cytosolic mtDNA-dependent activation of cGAS–STING inflammation and ameliorated deterioration of neurological function in old mito-QC reporter mice.^29^ Additionally, Parkin-deficient Drosophila melanogaster mutant’s exhibit disturbed mitochondria morphology together with their dysfunctional dynamics, which can be suppressed by loss of STING. This suggests feedback of STING signalling on mitochondria integrity.^28^ In a model of chronic mitochondrial stress, cross-breeding Parkin^−/−^ mice with the “Mutator” mice (mice that express a proofreading-deficient mtDNA polymerase (PolG) and accumulate mtDNA mutations) caused dopaminergic neuron degeneration and motor defects.^231^ Similarly, constitutively active STING was associated with DA neuron degeneration and promotion of neuroinflammation in mice,^45^ suggesting that the accrual of dysfunctional mitochondria may be necessary for the release of mtDNA to drive cGAS–STING-associated inflammation in PD. According to these observations, Borsche et al demonstrated elevated levels of both serum IL-6 and circulating cell-free mtDNA in patients biallelic for either Parkin or PINK1 gene mutations, implicating inflammation in Parkinson’s disease.^232^ This may suggest an interplay between mitochondrial stress and STING signalling in the pathology of PD.^190^ This is an attractive hypothesis, if true, the inhibition of the cGAS–STING pathway or finding ways to limit mitochondria pathology and release of mtDAMPs, especially mtDNA into the cytoplasm or circulation, may have therapeutic value in PD.^218^

Other mutations which were linked to PD occur in leucine-rich repeat kinase 2 (LRRK2, PARK8) as a cause of hereditary PD. Mutation of LRRK2 accompanied by impaired basal mitophagy achieves the accumulation of damaged mitochondria in the brain of the PD mouse model.^233^ In macrophages with LRRK2 knockout, aberrant increase in Drp1-associated mitochondrial fission as well as excessive oxidative stress result in chronic activation of the cGAS–STING signalling along with increased expression of ISGs.^43^ Overexpression of Drp1, in the preclinical models of optic atrophy 1 ablation, causes hyper-fragmentation of mitochondria and giant mtDNA nucleoids outside the mitochondria that are capable of eliciting the immune response.^234^ Thus, mtDNA–driven inflammation was also associated with altered regulation of mitochondrial dynamics.^235^,^236^ Circulating mtDNA is increasingly acknowledged as a pivotal mediator linking mitochondrial dysfunction to inflammation.^112^,^144^,^235^,^237^ When released from damaged mitochondria, cytosolic mtDNA engages the cGAS–STING pathway. Accordingly, stimulation of autophagy can also decrease the load of cytoplasmic DNA and may provide therapeutic benefits.^238^,^239^ Similarly, subtle regulation of mitochondrial dynamics could be a possible therapeutic strategy to relieve inflammatory stress and thus alleviate PD pathology.

## α-syn – a New Player in cGAS–STING Activation

Clearance of dysfunctional mitochondria may also be affected by α-syn – a central component to the pathogenesis of PD.^216^ α-syn aggregates engage both innate and adaptive inflammatory responses in CNS (gliosis, increased microglia antigen presentation, T cell infiltration, increased pro-inflammatory cytokines and chemokines), similar to classic DAMPs.^240–245^ Many studies implicate PRR-based mechanisms, including TLRs and NLRs signalling, as mediators of neuroinflammation in α-synucleinopathies.^241–243^,^246^,^247^ A relatively new issue is the α-syn–induced stimulation of the cGAS–STING signalling pathway. Pathologic α-syn aggregates evoke oxidative and nitrosative stress, which may entail genomic and mitochondrial DNA damage.^44^ The cGAS–STING pathway axis can be aberrantly stimulated by both mtDNA, as well as by genomic DNA in the context of damage to the cell nucleus, particularly when micronucleation and DNA breaks are present.^44^,^248^,^249^ Increasing evidence suggests that DNA damage and repair deficiency may be the direct mechanism involved in the pathophysiology of PD.^250–252^ Numerous studies revealed a significant up-regulation of the phosphorylated form of H2A Histone Family Member X (H2AX), γ-H2AX, a sensitive molecular marker of DNA double-strand breaks (DSBs), in PD, which type of damage was included not only DA neurons but also microglia.^253^,^254^ The study of Milanese demonstrated accumulation of DNA damage together with activation of the DNA damage response (DDR) in two different α-synucleinopathy PD mouse models. Interestingly, the observed accumulation of DSBs has been the initiating lesion of neurotoxicity, which preceded the onset of the motor phenotype and DA neuron degeneration.^253^ Pathogenic α-syn can damage DNA indirectly through ROS generation (α-syn aggregates localize to mitochondria and compromise respiratory chain components, resulting in mitochondrial dysfunction together with massive production of harmful ROS^255^) or directly cleave DNA, leading to DNA strand breaks.^256^,^257^ In neurons, α-syn induces DSBs in genomic DNA due to increased nitrosative stress.^258^ Recent research provides evidence that α-syn aggregates (intrastriatal α-syn performed fibril, α-syn-PFF) induce DSBs in microglial, genomic DNA, which in turn stimulates the STING-dependent IFN-I response resulting ultimately in inflammation that precedes neurodegeneration.^44^ Furthermore, the same authors provided evidence that genetic knockout of STING was neuroprotective in this α-syn-PFF model of PD. Mice without functional STING showed reduced inflammation, motor deficits, α-syn accumulation and DA neuron loss.^44^ Interestingly, there is also biochemical evidence that STING protein expression is up-regulated in the SNpc of PD patients, and this stimulation is correlated with the deposition of pathological α-syn.^44^ From these data, the authors suggest that stimulation of cGAS–STING pathway may exacerbate pathologic neuroinflammation and mediate neurodegeneration arising from nigrostriatal α-synucleinopathies, including idiopathic PD.^44^,^259^ Additionally, a study by Szego et al demonstrated that the chronic activation of STING was sufficient to cause neurodegeneration. In a mouse model that expresses the constitutively active STING variant N153S, researchers observed degeneration of DA neurons, a lower density of dopaminergic axon terminals and the concentration of dopamine in the striatum, α-syn pathology and a lower density of synaptic puncta, and neuroinflammation.^45^ Moreover, the histological evidence shows that the cGAS–STING pathway appears to be activated in endothelial and neural cells in multiple sclerosis, Alzheimer’s disease, Parkinson’s disease, and ALS. Together with the in vitro data, this suggests that the cGAS–STING pathway might be activated via α-syn–induced generation and accumulation of genomic DNA damage, as well as by mitochondrial stress and DNA leakage, resulting in downstream neuroinflammation.^260^

Moreover, α-syn–evoked Parkin deficiency may also lead to increased levels of oxidative genomic and mtDNA damage.^261^ There is evidence that Parkin protects nuclear DNA and regulates nucleotide excision repair (NER) mechanism.^262^ Nuclear translocation of Parkin seems to be required to promote DNA repair.^263^ DNA damage is able to induce inflammatory responses in microglia, astrocytes, and neurons,^264^,^265^ and the innate immune response induced by the cGAS–STING pathway appears to play a major role.^256^ Thus, beside genomic and mitochondrial DNA damage generation, overexpression or pathogenic aggregation of α-syn can also cause DNA repair defects by affecting the expression of DNA repair proteins, including the above-mentioned Parkin as well MRE11 and APE1.^256^,^266^,^267^ APE1 (DNA (apurinic/apyrimidinic site) endonuclease 1) is a pivotal enzyme of one of the major DNA repair routes - base excision repair (BER) pathway, which mainly repairs oxidative DNA damage and the alkylation of bases and is the dominant DNA repair pathway in neurons.^268^ The loss of APE1 expression was observed in the α-syn-PFF mouse model compared to controls,^269^ which exacerbates the accumulation of oxidised DNA damage.^270^ It has been also demonstrated that α-syn overexpression can lead to reduced expression of double-strand break repair protein MRE11 (MRE11) in the SH-SY5Y cells, thus promoting DNA DSBs.^267^ Considering the role of MRE11 in recognizing DSBs, and initiating the downstream repair process via forming an MRN complex with RAD50 double strand break repair protein (RAD50) and nibrin 1 (NBS1), down-regulation of MRE11 may lead to DNA double-strand break repair (DSBR) defects.^271^ Thus, α-syn interfered with the DNA repair system in microglia leading to the accumulation of DNA damage, and this may also trigger pro-inflammatory and neurotoxic signals through stimulation of the cGAS–STING pathway. Given the potential deleterious effects of pro-inflammatory factors and pathogenic α-syn on astrocytic genomic DNA,^265^,^272^ DNA damage may be an important driver of astrocyte activation, contributing to neurotoxic inflammation in these glial cells.^273^,^274^ Inflammatory factors secreted by glial cells can also cause DNA damage in the neuronal genome.^256^,^265^ Recent evidence suggests that DNA damage in neurons is also an important source of brain inflammation, engaging the inflammatory signalling triggered by the cGAS–STING pathway.^250^ Summarizing, α-syn might be a key initiating factor of the cGAS-STING-mediated neuroinflammation and neurodegeneration (Table 1).

**Table 1:** Direct and Indirect Evidence Suggesting the Possibility of α-Syn to Induce the cGAS–STING Pathway Activity, and Thus Neuroinflammation

**α-syn–induced generation and accumulation of DNA damage**
**Summary**
α-syn induces DSBs in genomic DNA due to increased nitrosative stress; α-syn-PFF activates NOS leading to increase NO levels and DNA damage (increase the expression of serine139-phosphorylated gamma histone H2A.X (γH2A.X), a marker of DNA strand).^258^
α-syn cause DNA strand breaks and this effect is exacerbated by its oligomerisation or oxidation; α-syn binds to DNA and chromatin; α-syn requires nuclear localisation and chromatin association to induce genome damage (chromatin-bound oxidized α-syn induces strand breaks in neuronal genomes).^257^
α-syn induces DNA damage (DSBs accumulation) together with activation of the DDR (significant increase in γH2A.X, p53-binding protein 1 (53BP1) *foci*, and in phospho-ataxia telangiectasia mutated (p-ATM) kinase immunoreactivity in dopaminergic neurons). The toxic cascade leading to DNA damage involves oxidant stress and mitochondrial dysfunction. The accumulation of DSBs preceded the onset of the motor phenotype and DA degeneration.^253^
α-syn induce DSBs in microglial, genomic DNA (significant increase in γH2A.X).^44^
**α-syn–induced DNA repair system defects by affecting the expression of DNA repair proteins: MRE11, APE1 and Parkin**
Endogenous α-syn DSBR facilitates the DNA non-homologous end-joining reaction (NHEJ). Cytoplasmic aggregation of α-syn reduces its nuclear levels (loss of the function of nuclear α-syn), increases DSBs, and may contribute to programmed cell death. Lewy inclusion-containing neurons in mouse model and human-derived patient tissue demonstrate increased DSBs levels.^266^
Overexpression of α-syn leads to impaired DNA repair and cellular senescence. α-syn overexpression led to cellular senescence with activation of the p53 pathway and DNA damage response (DDR). α-syn overexpression reduces the expression of MRE11, a key component of the DSBR system. Accumulation of p(S129)-α-syn is accompanied by an increase in DSBs DNA in α-syn transgenic mice.^267^
α-syn-PFF inclusions into the mouse olfactory bulb/anterior olfactory nucleus (OB/AON) elicit a modest decrease in the expression of APE1 (the multipurpose DNA repair/redox protein) in the brains of only male mice. Similarly, only men with Lewy body disorders display lower APE1 expression in the OB and amygdala.^269^
α-syn–evoked Parkin deficiency may result in increased levels of oxidative genomic and mtDNA damage. Endogenous Parkin is associated physically with mtDNA and co-associates with TFAM. Parkin protects mtDNA from oxidative damage and stimulates mtDNA repair.^261^
Parkin is essential for optimal repair of DNA damage. Parkin interacts with the proliferating cell nuclear antigen (PCNA), a protein that coordinates DNA excision repair, thus promoting DNA repair. Parkin-deficient mice show increased 8-oxoguanine in the cerebral cortex, and Parkin promotes both base and NER in cultured cells.^262^
Parkin promotes DNA repair and protects against genotoxicity. Parkin reduces DNA damage induced by UV irradiation. DNA damage induces nuclear translocation of Parkin, leading to the PCNA interaction and possibly other nuclear proteins involved in DNA excision repair. Nuclear localized Parkin promotes DNA excision repair.^263^
**α-syn–induced Parkin dysfunction disturbs the mitochondrial network homeostasis, leading to mitochondrial stress**
α-syn–induces oxidative/nitrosative stress leading to S-nitrosylation and inactivation of Parkin; the posttranslational modification of Parkin induces the elevation of Parkin autoubiquitination and degradation of this protein. Decrease Parkin levels result in cell death, whereas Parkin overexpression protects against α-syn toxicity.^5^
α-syn–evoked Parkin down-regulation (reduced Parkin levels in mitochondria and decreased ubiquitination of mitochondrial proteins) promotes mitochondrial dysfunction (depolarization of the mitochondrial membrane, elevated synthesis of the mitochondrial superoxide anion, decrease in cellular ATP level), impairs mitochondrial biosynthesis (reduction in PGC-1α protein levels), and disturbs the mitophagy process in neuronal cells, leading to an overall breakdown of mitochondrial homeostasis and the accumulation of defective mitochondria. Parkin overexpression has a protective effect on α-syn-induced mitochondrial dysfunction.^7^
**α-syn induces the cGAS–STING pathway activation leading to neuroinflammation**
α-syn aggregates induce DSBs in microglial, genomic DNA, which in turn stimulates the cGAS–STING-dependent IFN-I response resulting ultimately in inflammation that precedes dopaminergic neurodegeneration in a model of idiopathic PD; genetic knockout of STING is a neuroprotective in α-Syn-PFF model of PD, reducing inflammation, motor deficits, α-syn accumulation and DA neuron loss; STING protein expression is up-regulated in the SNpc of PD patients and this stimulation is correlated with the deposition of pathological α-syn (protein level of STING correlated linearly with the amount of p(S129)-α-syn among the PD tissue samples).^44^
**cGAS–STING pathway as an important mediator of α-syn pathology in PD**
Constitutively active STING causes strong neuroinflammatory phenotype consistent with systemic inflammation, degeneration of DA neurons, decrease in the both density of DA axon terminals and the concentration of dopamine in the striatum together with increase in the concentration of dopamine metabolites; constitutively active STING causes α-syn pathology in the striatum and substantia nigra (increase the both ratio of p(S129)-α-syn/α-syn and the ratio of Triton X-100 insoluble/soluble α-syn, increase the number of cells with thioflavin S (ThioS)-positive inclusions in the striatum) and synaptic defects in the striatum (lower density of both synaptic and post-synaptic puncta); chronic STING activation via downstream signalling by IFN-I or inflammasome activation (differential activation of IFN-dependent and IFN-independent signalling between brain regions) is sufficient for degeneration DA neurons and course neurodegeneration.^45^

## Limitations

Despite intensive research conducted in the last few years, several critical questions remain to be answered. The major outstanding questions:

1. To what extent the α-syn–induced neuroinflammation is the result of stimulation of the cGAS–STING pathway and to what extent do other signalling cascades, which recognize endogenous danger signals (DAMPs) operating in the brain, including TLR (TLR9, TLR4) and NLR signalling (NLRP3)? What are the relative contributions of these pathways to the modulation of inflammatory responses as a consequence of α-syn–induced stress, as compared to the cGAS–STING pathway?
2. To what extent α-syn is able to activate the cGAS–STING pathway in neuronal cells and glial cells, respectively? What are the relative proportions of neurons, astrocytes and microglia in different brain regions capable of activating the α-syn–evoked cGAS–STING pathway? It appears that genetic activation of cGAS–STING in different cell types will be required to determine the contributions of STING activation within glial cells and neurons.
3. Does STING–induced neurodegeneration of dopaminergic neurons could initiate degeneration of further neuron populations? Are some brain regions and neuron populations more susceptible to STING–induced damage than others? An area which merits further investigation is the analysis of the individual immune response of different brain regions, which could provide new information about the differential susceptibility of the various brain regions to STING–evoked damages, as a result of stress induced by α-syn.
4. While activation of immunostimulatory pathways is required for the normal functioning of the brain, persistent engagement of these responses can prove detrimental. What must be the magnitude and scale of α–syn–induced pathology, which is necessary for cGAS–STING pathway activation to such an extent that to tip the balance towards neuroinflammation?
5. In response to various stresses in dynamic microenvironments, cGAS–STING can stimulate the process of apoptosis, autophagy/mitophagy, necroptosis, pyroptosis, and ferroptosis. Which type of cell death is triggered due to α–syn–induced cGAS–STING pathway stimulation?
6. What is the exact sequence of molecular events linking α-syn oligomerization/aggregation to Parkin–mediated mitochondrial dysfunction and ultimately cGAS–STING activation? This field needs to be more rigorously characterized. Elucidation of the molecular mechanisms underlying immune activation in the CNS would yield valuable clues to develop novel therapeutics.
7. Which processes in the cascade of α-syn–induced pathological events leading to inflammation are crucial in the pathogenesis of PD? At what stage of pathological molecular events would therapeutic intervention be effective? (Parkin dysfunction/mitochondria damage/DNA damage and repair abnormalities/cGAS–STING overstimulation?). Clarifying the molecular events in the α-syn–induced cascade of pathological events and the relationships between them might open new avenues for understanding the pathogenesis of PD and provide fertile ground for future drug development; identifying a potential point of therapeutic intervention would be possible.
8. One of the mtDAMPS, cytosolic mtDNA, triggers inflammatory responses. Proposed are several routes by which mtDNA is released into the cytosol to mediate inflammatory responses. It is not well elucidated how mtDNA leaks exactly into the cytosol and whether mtDNA present in the cytosol could be a therapeutic target.
9. How effective and afford neuroprotection will be the action on the deregulated cGAS–STING itself (inhibitors of STING, cGAS–targeted inhibitors), and to what extent would multi-point therapeutic intervention (in addition to cGAS–STING inhibition) including for example Parkin–targeted activators, mitochondrial therapies, and therapeutic strategies targeting DNA damage repair would be necessary in the case of α-syn–induced neurotoxicity?

Additionally, the studies of the interplay between cells of the blood-brain barrier and the component cells of the brain, which comprise the neurovascular unit, could prove helpful in the analysis of α-syn–induced pathology, and lead to the discovery of the potential communication channels between the brain and periphery, which may be targeted to identification of potential biomarkers of PD (blood- or CSF-based biomarkers).

## Conclusions and Future Directions

**Figure 1:**
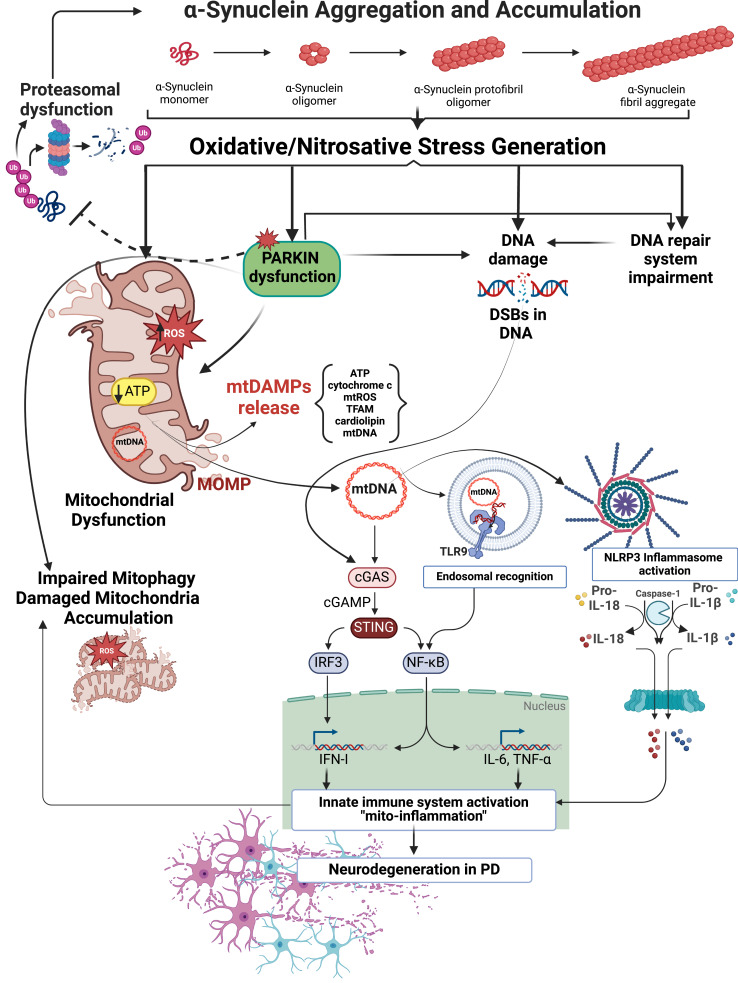
Proposed mechanisms of α-syn neurotoxicity in PD. Pathological α-syn aggregates evoke oxidative/nitrosative stress leading to mitochondrial damage, S-nitrosylation and inactivation of E3 ubiquitin ligase Parkin, generation and accumulation of DNA damage, and DNA repair system impairment. α-syn–evoked Parkin down-regulation promotes mitochondrial dysfunction and impairment mitophagy in neuronal cells, which results in the accumulation of damaged mitochondria. Moreover, α-syn–induced Parkin dysfunction disturbs proteasomal degradation of misfolded proteins leading to the accumulation of defective proteins, including α-syn. Parkin deregulation may also lead to exacerbation of oxidative genomic DNA damages induced by α-syn itself. Parkin deficiency, combined with mitochondrial damage and mtDAMPs release, including mtDNA, might modulate the inflammatory response by stimulating NLRP3, triggering endosomal TLR9 receptors, or by activation of the DNA-induced cGAS–STING pathway. NLRP3 stimulation elicits robust caspase-1 activation and consequent proteolytic maturation of IL-1β and IL-18. mtDNA can be bound by TLR9 in the endosome promoting the activation of a MyD88 pathway that ultimately leads to the expression of pro-inflammatory cytokines. In turn, the induction of cGAS–STING downstream effectors such as IRF3 and NF-κB turns on robust transcription of IFN-I and a number of pro-inflammatory factors (IL-6 and TNF-α) to enhance innate immune response. The cGAS–STING pathway might be also stimulated by α-syn–induced genomic DNA damages in the context of nuclear dysfunction, particularly when micronucleation and DNA breaks are present. Finally, evoked by α-syn the innate immune system activation and chronic inflammation (“mito-inflammation”) may promote neurodegeneration in PD and exacerbate mitochondrial damage, thus generating a vicious cycle of neurotoxic events. This Figure was partially created using BioRender dynamic assets and retrieved from https://app.biorender.com/biorender-templates.

**Figure 2:**
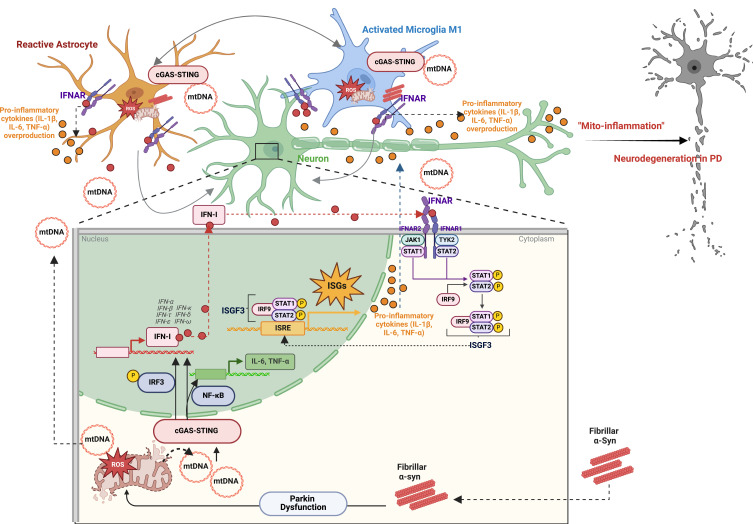
Schematic representation of the α-syn–induced events (Parkin-mediated mitochondrial dysfunction and excessive stimulation of cGAS–STING pathway together with IFN-I signalling) contributing to neurodegeneration in PD. α-syn–induced Parkin dysfunction promotes mitochondrial failure and impairment mitophagy in neuronal cells, leading to the accumulation of damaged mitochondria and the release of the various mtDAMPs. The release of mtDAMPs, including mtDNA, can induce inflammatory responses by activating the innate immune system in neurons and glial cells. A portion of the released mtDNA binds to cGAS, activating the intracellular cGAS–STING pathway. Activation of cGAS–STING leads to the induction of downstream effectors such as IRF3 and NF-κB, triggering robust transcription of genes encoding IFN-I and other pro-inflammatory factors, including IL-6 and TNF-α. IFN-I, once secreted, acts through IFNAR on target cells (neurons, microglia and astrocytes), comprising IFNAR1 and IFNAR2 subunits, associated with TYK2 and JAK1, respectively. In turn, stimulation of TYK2 and JAK1 promotes the activation of STAT1 and STAT2 proteins, which bind to the IRF9 forming an ISGF3. The ISGF3 complex migrates to the nucleus and then by binding genes harboring the ISRE stimulates the transcription of numerous ISGs, including pro-inflammatory IL-1β, IL-6, and TNF-α. Additionally, mtDNA released by neuronal cells activates the cGAS–STING pathway in neighboring glial cells, thereby enhancing IFN-I–mediated immune responses. Prolonged overactivation of glial cells and excessive engagement of the cGAS–STING signaling can lead to chronic neuroinflammation, contributing to neurodegeneration. Long-term overstimulation of glia results in the overproduction and release of harmful stress–inducing pro-inflammatory factors, aggravating the inflammatory environment and inducing neuronal cell death and tissue damage, thereby promoting neurodegeneration in PD. Thus, the aberrant cGAS–STING-triggered inflammatory response to pathological α-syn may be one of the key mechanisms driving the progression of neurodegeneration in PD. This Figure was partially created using BioRender dynamic assets and retrieved from https://app.biorender.com/biorender-templates.

Taken together, in light of the available data, it is reasonable to conclude that α-syn–induced mitochondrial pathology has been consistently associated with the release of mtDAMPs, genomic and mitochondrial DNA damage with simultaneous impairment of the DNA repair system, which may collectively cause cGAS–STING–driven neuroinflammation ultimately resulting in progressive neurodegeneration (Figures 1 and 2). In particular, emerging evidence suggests that mitochondria play an important role in the control of inflammatory responses, offering a unique platform for mtDAMPs redistribution, PRRs signalling and inflammation in the context of failing adaptation to cellular stress. Considering the important role of mitochondria in pro-inflammatory processes, we suggest that therapeutic manipulations aiming to prevent mitochondrial damage, and/or disruption of mitophagy may prevent mtDAMPs–induced cGAS–STING activation and detrimental “mito-inflammation”. Triggered by α-syn, the cGAS–STING–driven “mito-inflammation” may be a key mechanism of progressive neurodegeneration in PD, opening doors to new therapeutic approaches targeting the innate immune response in PD. We hypothesise that the cGAS–STING pathway can be activated during the progression of PD and through this activation a neurotoxic environment could be created. Therefore, cGAS or STING inhibition may serve as a viable therapeutic approach for slowing down the progression of PD. To investigate the mechanisms, genetically modified animals lacking either cGAS or STING expression crossed with α-syn overexpression lines in order to uncover the role of α-syn-dependent pathways could be a useful research tool. A better understanding of the innate immune signalling events taking place during PD onset and progression could reveal new possible therapeutic targets.

## Abbreviations

AAV, adeno-associated virus; AIM2, absent in melanoma-2; ALS, amyotrophic lateral sclerosis; AMBRA1, autophagy and beclin 1 regulator 1; AMPK, AMP-activated protein kinase; AP-1, activator protein 1; APE1, DNA (apurinic/apyrimidinic site) endonuclease 1; ASC, apoptosis-associated speck-like protein containing a C-terminal caspase recruitment domain; ATM, ataxia telangiectasia mutated kinase; ATP, adenosine triphosphate; BAK1, Bcl-2 antagonist/killer 1; BAX, Bcl-2-associated X; Bcl-2, B-cell lymphoma-2; BER pathway, base excision repair pathway; CCL20, C-C Motif Chemokine Ligand 20; cGAMP, 2′3′- cyclic GMP-AMP; cGAS, cyclic GMP-AMP synthase; cGAS–STING, cyclic GMP-AMP synthase (cGAS)–stimulator of interferon genes (STING); CNS, central nervous system; COPII, coatomer protein complex II; CXCL10, C-X-C Motif Chemokine Ligand 10; CXCL2, C-X-C Motif Chemokine Ligand 2; DA, dopaminergic; DAMPs, damage-associated molecular patterns; DDR, DNA damage response; DLB, dementia with Lewy bodies; Drp1, dynamin-related protein 1; DSBR, DNA double-strand break repair; DSBs, double-strand breaks; dsDNA, double-stranded DNA; ER, endoplasmic reticulum; ERGIC, ER–Golgi intermediate compartment; ESCs, embryonic stem cells; ETC, mitochondrial electron transport chain; EVs, extracellular vesicles; Fis1, mitochondrial fission protein 1; GBA1, glucocerebrosidase beta 1; GTP, guanosine triphosphate; H2AX, H2A Histone Family Member X; H_2_O_2_, hydrogen peroxide; HIF-1, hypoxia-inducible factor 1; HMGB, high mobility group box; IFI16, interferon-inducible protein 16; IFNAR, type I interferon receptor/or interferon-α/β receptor; IFNAR1, interferon-α/β receptor alpha chain; IFNAR2, interferon-α/β receptor beta chain; IFN-I, type I interferons; IFN-α, interferon-alpha; IFN-β, interferon-beta; IKK, IκB kinase; IL-18, interleukin-18; IL-1β, interleukin-1β; IL-6, interleukin-6; iNOS, inducible nitric oxide synthase; iPSCs, induced pluripotent stem cells; IRF3, interferon regulatory factor 3; IRF9, interferon regulatory factor 9; ISGF3, interferon-stimulated gene factor-3; ISGs, interferon-stimulated genes; ISRE, interferon-sensitive response element; JAK1, Janus kinase 1; LB, Lewy bodies; LLPS, liquid-liquid phase separation; LPS, lipopolysaccharide; LRRK2, leucine-rich repeat kinase 2; MAPK, mitogen-activated protein kinase; MAPK/JNK, mitogen-activated protein kinase/c-Jun N-terminal kinase; MDVs, mitochondria-derived vesicles; Mff, mitochondrial fission factor; Mfn1, mitofusin 1; Mfn2, mitofusin 2; MOMP, mitochondrial outer membrane permeabilisation; mPTP, mitochondrial permeability transition pore; MQC, mitochondrial quality control; MRE11, double-strand break repair protein MRE11; MSA, multiple system atrophy; mtDAMPs, mitochondrial-derived damage-associated molecular patterns; mtDNA, mitochondrial DNA; mTOR, mammalian target of rapamycin; mTORC1, mTOR complex 1; mtROS, mitochondrial-derived reactive oxygen species; MyD88, myeloid differentiation primary response 88; NBS1, nibrin 1; NDP52, nuclear dot protein 52; NER, nucleotide excision repair; NFAT, nuclear factor of activated T-cells; NF-κB, nuclear factor κB; NHEJ, non-homologous end-joining reaction; NLR, (NOD)-like receptor; NLRP3, nucleotide-binding oligomerization domain (NOD)-like receptor family pyrin domain containing protein 3; NLRs, NOD-like receptors; NO, nitric oxide; NOS, nitric oxide synthase; NOX, mitochondrial NADPH oxidase; NPCs, neural lineage progenitor cells; NRF2, nuclear factor-erythroid 2 related factor 2; OB, human olfactory bulb; OB/AON, olfactory bulb/anterior olfactory nucleus; Opa-1, optical atrophy 1; OPTN, optineurin; PAF, pure autonomic failure; PAMPs, pathogen-associated molecular patterns; Panx1, Pannexin 1; PARIS, Parkin-interacting substrate; PARK2, Parkinson Disease Protein 2; PCNA, proliferating cell nuclear antigen; PD, Parkinson’s disease; pDCs, plasmacytoid dendritic cells; PGC-1, peroxisome proliferator-activated receptor gamma coactivator 1; PINK1, PTEN-induced putative kinase 1; PKC/MAPK, protein kinase C/mitogen-activated protein kinase; PRC, PGC-1-related coactivator; PRRs, pattern-recognition receptors; PYHIN, pyrin and hematopoietic interferon-inducible nuclear (HIN) domain; RAD50, RAD50 double strand break repair protein; RAGE, receptor for advanced glycation endproducts; RBD, REM sleep behaviour disorder; RNS, reactive nitrogen species; ROS, reactive oxygen species; SAR1A, secretion associated, Ras related GTPase 1A; SEC24C, SEC24-related protein C; SNpc, substantia nigra pars compacta; STAT1 and STAT2, signal transducer and activator of transcription 1 and 2; STIM1, stromal interaction molecule 1; STING, stimulator of interferon genes; TBK1, TANK-binding kinase 1; TFAM, mitochondrial transcription factor A; ThioS, thioflavin S; TLR9, Toll-like receptor 9; TLRs, Toll-like receptors; TNFR, tumour necrosis factor-α (TNF-α) receptor; TNF-α, tumour necrosis factor-α; TYK2, Tyrosine kinase 2; ULK1, Unc-51-like kinase 1; UPRmt, mitochondrial unfolded protein response; VDAC1, voltage-dependent anion channel 1; α-syn, alpha-synuclein; α-syn-PFF, alpha-synuclein preformed fibrils; γH2A.X, serine139-phosphorylated gamma histone H2A.X; 53BP1, p53-binding protein 1.
